# Postnatal development of extracellular matrix and vascular function in small arteries of the rat

**DOI:** 10.3389/fphar.2023.1210128

**Published:** 2023-08-15

**Authors:** Zahra Nourian, Kwangseok Hong, Min Li, Jorge A. Castorena-Gonzalez, Luis A. Martinez-Lemus, Philip S. Clifford, Gerald A. Meininger, Michael A. Hill

**Affiliations:** ^1^ Dalton Cardiovascular Research Center, Columbia, MO, United States; ^2^ Department of Medical Pharmacology and Physiology and University of Missouri, Columbia, MO, United States; ^3^ Department of Physical Education, College of Education, Chung-Ang University, Seoul, Republic of Korea; ^4^ Department of Pharmacology, Tulane University School of Medicine, New Orleans, LA, United States; ^5^ College of Applied Health Sciences, University of Illinois at Chicago, Chicago, IL, United States

**Keywords:** extracellular matrix proteins, elastin, small artery development, myogenic reactivity, endothelial function

## Abstract

**Introduction:** Vascular extracellular matrix (ECM) is dominated by elastic fibers (elastin with fibrillin-rich microfibrils) and collagens. Current understanding of ECM protein development largely comes from studies of conduit vessels (e.g., aorta) while resistance vessel data are sparse. With an emphasis on elastin, we examined whether changes in postnatal expression of arteriolar wall ECM would correlate with development of local vasoregulatory mechanisms such as the myogenic response and endothelium-dependent dilation.

**Methods:** Rat cerebral and mesenteric arteries were isolated at ages 3, 7, 11, 14, 19 days, 2 months, and 2 years. Using qPCR mRNA expression patterns were examined for elastin, collagen types I, II, III, IV, fibrillin-1, and -2, lysyl oxidase (LOX), and transglutaminase 2.

**Results:** Elastin, LOX and fibrillar collagens I and III mRNA peaked at day 11–14 in both vasculatures before declining at later time-points. 3D confocal imaging for elastin showed continuous remodeling in the adventitia and the internal elastic lamina for both cerebral and mesenteric vessels. Myogenic responsiveness in cannulated cerebral arteries was detectable at day 3 with constriction shifted to higher intraluminal pressures by day 19. Myogenic responsiveness of mesenteric vessels appeared fully developed by day 3. Functional studies were performed to investigate developmental changes in endothelial-dependent dilation. Endothelial-dependent dilation to acetylcholine was less at day 3 compared to day 19 and at day 3 lacked an endothelial-derived hyperpolarizing factor component that was evident at day 19.

**Conclusion:** Collectively, in the rat small artery structural remodeling and aspects of functional control continue to develop in the immediate postnatal period.

## Introduction

Vascular extracellular matrix (ECM) contains an array of structural matrix proteins. These include elastin, fibrillar collagens as well as adhesive glycoproteins such as, fibronectin, and basement membrane components including Type IV collagen and laminins that are synthesized by vascular smooth muscle cells (VSMC), fibroblasts and endothelial cells (Hallmann et al., 2005; Wagenseil and Mecham, 2009) Collectively these macromolecules are vital for tissue mechanical integrity and are importantly linked to cell movement and cell-matrix adhesive interactions (Kelleher et al., 2004; Hynes and Naba, 2012) and together contribute to the overall mechanical function of large elastic vessels, such as aorta (Wagenseil, 2011), and small muscular arteries. Of these ECM proteins, changes in elastin, in particular, have been shown to play an important role in aging and vascular disease (Duca et al., 2016; Le Page et al., 2019; Yanagisawa and Wagenseil, 2020; Adeva-Andany et al., 2021).

Elastic fibers, the functional form of elastin, represent one of the most important ECM protein complexes linked to the extensibility and recoil properties of the blood vessel wall (Vrhovski and Weiss, 1998). Elastic fibers consist of two distinct domains. An inner core that is composed of elastin and a surrounding layer composed of microfibrils (Kielty et al., 2002; Wagenseil and Mecham, 2007). Elastin is genetically encoded by a single gene in mammals and is secreted as a 60–70 KDa soluble monomer (tropoelastin). Tropoelastin contains alternating hydrophobic amino acids and hydrophilic cross-linking domains (Gray et al., 1973; Brown-Augsburger et al., 1995). Once secreted, tropoelastin is crossed linked in a process initiated by lysyl oxidase (LOX, a copper-dependent amine oxidase) to produce the large insoluble form of elastin (Sandberg et al., 1971; Rodriguez et al., 2008). Co-incident with this, microfibrillar proteins, including fibrillin and fibulins, associate with the polymerized elastin to form functional the elastic fibers present in the vascular wall (Wagenseil and Mecham, 2007). As a result of the relatively stable nature of elastin and its integral relationship with the vascular wall an understanding of its function requires knowledge of how it changes during development and aging.

Collagens are also a large family of ECM proteins in the arterial wall which undergo age-related changes in their synthesis and assembly (Ponticos and Smith, 2014). In mouse aorta collagen types I, III, IV, V, and VI are expressed at significant levels. Amongst these collagen types I, III, and V are located in the vascular adventitia and are thought to provide the blood vessel with strength and stability during exposure to mechanical stress as imposed by high intravascular pressure (Kelleher et al., 2004). Collagen type IV, in contrast, is a major components of the vascular basement membrane located at the basal layer of the endothelial cells and surrounding smooth muscle cells (Hallmann et al., 2005). Thus, the temporal patterns of expression as well as the spatial distribution and organization of ECM proteins are important areas for investigation in order to better understand development, aging and changes that accompany vascular disease.

In studies, using 3D confocal microscopy, we and others have demonstrated a complex arrangement of elastin fibers in the walls of resistance arteries (Arribas et al., 2007; Clifford et al., 2011; Bloksgaard et al., 2015; Hill et al., 2016). Further, marked differences in elastin content and organization/distribution were observed in small cranial arteries compared to those of either mesentery or cremaster muscle in young adult rats (Clifford et al., 2011). As indicated above, our current knowledge of the developmental patterns of elastin expression, and other ECM proteins, comes mostly from the study of large blood vessels (Kelleher et al., 2004), as well as tissues such as lung (Wendel et al., 2000; Shi et al., 2007) and skin (Pasquali-Ronchetti and Baccarani-Contri, 1997; Baldwin et al., 2013). Equivalent data from resistance vessels and the microvasculature are, however, currently lacking. Given the marked heterogeneity we have observed in elastin distribution both between and within different vascular beds (Clifford et al., 2011; Hill et al., 2016), it is unclear whether data obtained in conduit vessels can necessarily be extrapolated to the resistance vasculature. Further, it is unknown whether such heterogeneity is of functional significance.

The present studies aimed to develop a more detailed understanding of the developmental and age-related changes in the expression of arteriolar wall ECM proteins. We chose rat cerebral and mesenteric resistance arteries for study as previous studies had demonstrated marked differences in content and organization of elastin in these vessels (Clifford et al., 2011) which we hypotheszied might also be reflected in other ECM proteins. Additional studies using 3D confocal imaging were undertaken to determine age-related changes in the patterns of adventitial and IEL elastin organization. A final aim was to determine if postnatal changes in ECM protein expression and structure are associated with changes small artery function as shown by myogenic reactivity and endothelial-dependent relaxation. This latter aim was driven by previous studies having been confined to large animal experimental models (Zellers and Vanhoutte, 1991; Nankervis et al., 2001a; Nankervis et al., 2001b; Reber et al., 2002) or using large conduit vessels from rodents such as mice aorta or carotid arteries (Flavahan and Flavahan, 2014).

## Materials and methods

### Tissue isolation and vessel RNA purification

All experiments and protocols were conducted according to protocols approved by the Animal Care and Use Committee, University of Missouri, United States. The studies used neonatal Sprague-Dawley rats at 3, 7, 11, 14, and 19 days and adults at 2 months and 2 years of age. Prior to surgical harvesting of vessels, rats were maintained in an accredited animal facility with 12:12 h light: dark cycle, controlled environmental conditions and food and water *ad libitum*. For vessel isolation neonatal rats were removed from their mothers immediately prior to an experiment and anesthetized with sodium pentobarbital (Nembutal, 35 mg/kg body weight) given by an intraperitoneal injection. Mature rats were similarly anesthetized with sodium pentobarbital (100 mg/kg body weight). On demonstration of a surgical plane of anesthesia mesenteric tissues were surgically removed and placed in a cooled (4°C) chamber containing dissection buffer (in mmol/L) 3 MOPS, 145 NaCl, 5 KCl, 2.5 CaCl_2_, 1 MgSO_4_, 1 NaH_2_PO_4_, 0.02 EDTA, 2 pyruvate, 5 glucose plus 1% bovine serum albumin). Following euthanasia by anesthetic overdose, a craniotomy was performed, the brain removed and placed in a cooled dissection chamber. Small cerebral arteries were isolated from the Circle of Willis, while mesenteric arteries were dissected from 2^nd^ and 3^rd^ order branching vessels. Isolated cerebral and mesenteric arteries were rapidly subjected to total RNA purification using a Melt Total Nucleic Acid isolation system kit (Life Technologies, CA, United States) according to the manufacturer’s instructions. Concentration and purity of RNA for each sample was determined by UV absorbance using a ND-1000 spectrophotometer (Nanodrop, Fisher Thermo, Wilmington, DE, United States). Equal amounts of total vessel RNA extract were then reverse-transcribed into single strand cDNA using a Superscript III First-Strand synthesis system (Life Technologies, CA) according to the manufacturer’s instructions. The resulting cDNA template from each sample was subsequently used for real-time quantitative PCR (qPCR) analysis.

Sex of rats used in the functional studies at ages 3 and 19 days was confirmed genetically via detection of the *SRY* gene. In mammals, the sex-determining region Y (*SRY*) gene, also known as Testis-determining factor (TDF) located on the Y chromosome, is the only gene necessary and sufficient for testis determination (Oh and Lau, 2006; Ngun et al., 2011). To detect the *SRY* gene, all cDNA samples were amplified with forward (5’-TGG GAT TCT GTT GAG CCA ACT-3’) and reverse (5’-GCG CCC CAT GAA TGC AT-3’) primers by end-point PCR. Using this approach, we determined 60% of the postnatal rats to be male and 40% female.

### Real-time quantitative PCR for mRNA coding for matrix and matrix-associated proteins

Relative mRNA expression levels for elastin, fibrillin-1, fibrillin-2, lysyl oxidase (LOX) and tissue transglutaminase 2 (t-TG) were initially determined using a Mastercycler EP Realplex^2^ system (Eppendorf-North America, NY, United States) and the fluorescent dye SYBER Green (see Supplementary Table S1 for sequences of primer sets). In these assays, real-time PCR (qPCR) reactions were performed in triplicate on cDNAs prepared from each sample (n = 4–5) using KAPA SYBER FAST qPCR Kit Master mix (KAPA Biosystems, Woburn, MA, United States). SYBER Green reaction volume/well contained 20 µL: 10 µL of master mix, 1 µL of each forward and reverse primer (5 µM), 1 µL of cDNA templates and the remainder DNase-free water. PCR conditions were as follows: pre-heating at 95°C for 2 min, 40 cycles of two-step cycling of denaturation at 95°C for 3 s and anneal/extend steps of 25 s at 56°C. For each PCR run, no enzyme and no template controls were included to test for contamination of assay reagents. At the completion of PCR melt curve analysis was performed to demonstrate PCR product purity.

To verify and extend the initial elastin data, relative mRNA expression of elastin plus selected additional ECM proteins (collagen types 1, 2, 3, and 4, Emillin-1 were also determined using specific pre-loaded FAM-labeled TaqMan probes and primer sets (Life Technology, CA, United States) (see Supplementary Table S2 for details). QPCR reactions were performed for each sample (n = 4–5) in customized 96 well plates containing separate TaqMan assays in duplicate. Each well contained 10 µL TaqMan Fast advanced Master Mix (Life Technology, CA, United States), 1 µL of cDNA template and DNase-free water to reach a final volume of 20 µL. QPCR reactions were performed using FAM as the detector probe under the following conditions: pre-heating at 50°C for 2 min, denaturation at 95°C for 1 s and 40 cycles of amplification at 60°C for 20 s 18S rRNA was included in each qPCR experiment to verify the quality of RNA. β-actin and GAPDH TaqMan assays were also included to verify age-dependent stability of housekeeper genes expression. β-actin (Actb) expression used as a reference gene and changes in mRNA expression levels were calculated as the fold relative to elastin mRNA expression of a cerebral artery sample prepared from a 2-month-old rat. This sample was included as a calibrator for all SYBER and TaqMan qPCR assays. Data were collected and analyzed using Realplex software (Eppendorf-North America, NY, United States). Relative quantification was performed using the comparative threshold (Ct) method after determining the Ct values for reference gene (β-actin) and the target genes in each sample set according to the 2^−ΔΔCT^ method (Livak and Schmittgen, 2001).

### QPCR for mRNA coding for proteins associated with vasodilator pathways

**FIGURE 1:**
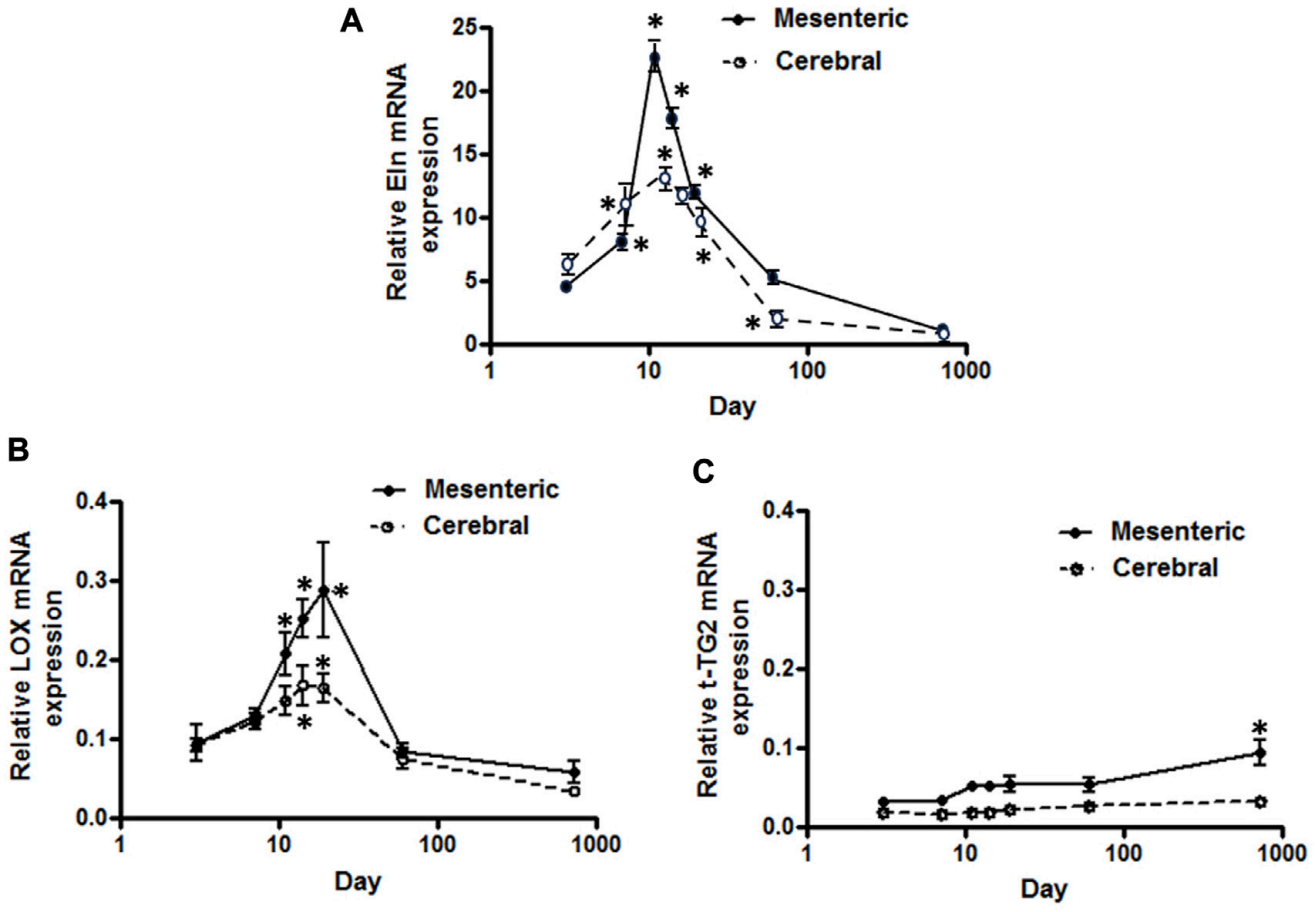
Developmental changes in elastin mRNA expression in rat cerebral vs. mesenteric arteries. Temporal relative mRNA expression profiles for **(A)** elastin **(B)** lysyl oxidase (LOX), and **(C)** transglutaminase 2 (t-TG2) in rat postnatal days 3,7,11,14, and 19, 2 month and 2 year old. X axis (age) is shown as a log scale. PCR data were obtained using SYBER Green. β-actin gene expression used as reference gene and changes in mRNA expression was calculated as fold changes relative to elastin expression of a calibrator, an arbitrary 2 month old cerebral artery sample. Results are shown for n = 4–5 separate experiments and are presented as mean ± SEM. Elastin and LOX mRNA levels show the same profile, rise significantly from postnatal day 3 to a peak at 11–19 day after which expression also significantly declines throughout life in both vessel types. t-TG2 gene expression profile, however, showed a relatively constant increase from day 3 throughout all time points studied with significant increase at 2 year old compared with 3 day in each vasculature. *p < 0.05 compared to day 3 for respective vessel type; one way ANOVA.

Real-time PCR reactions using the fluorescent dye SYBER Green were performed to determine the age-dependent changes in endothelial nitric oxide synthase (eNOS), prostacyclin synthase, and Ca^2+^-activated K^+^ channels including small (SK_Ca_), intermediate (IK_Ca_) and large (BK_Ca_) channels (See Supplementary Table S3 for details of primer sets). These mRNA targets were chosen due to their known involvement in coding for critical proteins underlying various vasodilator pathways. Experimental conditions for SYBER Green-based PCR were similar to those described earlier with the exception of using 42 cycles of two-step cycling of denaturation at 95°C for 3 s and anneal/extend steps of 25 s at 58°C. To calculate the relative fold mRNA expression for each of the chosen genes in cerebral and mesenteric vasculatures, 3- day-old samples have been chosen as calibrator separately. β-actin was used as a references gene for data normalization. Using GAPDH, as a second housekeeping gene, parallel calculations were performed to check validity of the β-actin data (see Supplementary, Figure 1).

### Elastin staining and 3D confocal microscopy

*In situ* elastin fibers within the arterial wall were identified and analyzed from images collected using 3D fluorescence confocal microscopy as previously described (Clifford et al., 2011). Segments of superior cerebellar and small mesenteric arteries from 3, 7, 11, 14, 19 days, 2 months and 2 years old rats were dissected and cannulated onto glass micropipettes mounted in a pressure myograph (Living Systems Instrumentation, VT, United States). The cannulated arteries of the neonatal animals were pressurized to 70% of systemic pressure previously reported for rats aged from birth to 1 month (Litchfield, 1958). Vessels from 2 month to 2 year old rats were pressurized to 70 mmHg. Vessels remained pressurized during all steps of tissue processing and throughout imaging procedures. Pressurized small artery segments were fixed in 2% paraformaldehyde (10 min), rinsed twice in phosphate-buffered saline (PBS) and permeabilized with 0.5% Triton X-100 followed by extensive washing with PBS. Vessels were then stained with 0.2 µM Alexa Fluor 633 Hydrazide (Life Technology A30634; excitation 633/emission 700 nm). This fluorochrome has been previously verified to preferentially binds to elastin (Clifford et al., 2011), Fluorescent nuclear staining was performed with 1 µM Yo-Pro-1 iodide (Life Technology Y3603; excitation 491/emission 509 nm). All dyes, paraformaldehyde, and Triton X-100 were diluted in physiological saline solution (PSS) containing (in mmol/L) 145 NaCl, 4.7 KCl, 2 CaCl_2_, 1 MgSO_4_, 1.2 NaH_2_PO_4_, 4 glucose, 2 pyruvate with 20 min incubation time at room temperature. 3D image data sets were collected using a Leica TCS-SP5 confocal microscope, equipped with a Leica ×63 water objective lens (Numerical Aperture 1.2). Z-plane image slices were collected at 0.5 µm intervals as previously described (Clifford et al., 2011).

### 3D imaging analysis of elastic fibers in vascular wall

Alexa Fluor 633 was excited with a HeNe laser at 633 nm to visualize fluorescent staining for elastin (Clifford et al., 2011). Analyses of elastin expression/distribution were performed to quantify and characterize IEL fenestrae using NIH Image (NIH, Bethesda, MD). Briefly, a representative region of interest (ROI) of approximately 60 × 120 µm of vessel image z-stacks was selected from each image to minimize the effects of vessel curvature. Cropped vessel stacks were then analyzed to identify and label the fenestrae. Total numbers of fenestrae and average area per fenestrae (µm^2^) within the ROI were computed for each vessel segment and compared across age-groups using GraphPad Prism.

### Functional assessment of mesenteric and cerebral arteries of 3 and 19 day-old rats

Small mesenteric and superior cerebellar arteries were isolated from Sprague-Dawley rats at 3 and 19 days of age. Isolated vessels were cannulated onto glass micropipettes and mounted in a 7 mL chamber of a cannulation stage as previously described (Hill et al., 2000). The micropipettes and cannulated mesenteric arteries contained a modified Kreb’s buffer containing (in mM/L: NaCl 112, Glucose 10.5, NaHCO_3_ 25.5, HEPES 10, MgSO_4_.7H2O 1.2, CaCl_2_.2H2O 2.5, KH_2_PO_4_ 1.2, KCl 4.7, adjust PH to 7.3). All solutions were maintained at 37°C during an experiment. The cannulation chamber was placed on an inverted microscope and continuously suffused with Kreb’s buffer (4 mL/min). As the physiological relevant range of mean arterial blood pressure for these two postnatal ages are between 20–80 mmHg (Litchfield, 1958), the cannulated arteries were initially pressurized to 20 mmHg without luminal flow. Pressurized arteries were gently stretched in the longitudinal direction such that while increasing intraluminal pressure from 5 to 50 mmHg lateral bowing did not occur. Arteries were then warmed to 37°C and equilibrated for approximately 1 h prior to functional assessment.

Following the equilibration period, pressure-diameter relationships were recorded across a pressure range of 5–50 mmHg in the absence of flow. After measurement of myogenic responsiveness, vessels were set to an intraluminal pressure of 30 mmHg and endothelial function assessed by responses to acetylcholine (ACh; 10^−8^M-10^−5^M). To evaluate components of ACh-induced dilation, concentration response curves were repeated in the presence of combinations of eNOS inhibitor (L-NAME, 100 µM), prostaglandin synthesis inhibitor (indomethacin, 10 µM), SK_Ca_ (apamin, 1 µM), and IK_Ca_ blockers (TRAM-34, 1 µM). Since the pressurized cerebral arteries developed differing levels of myogenic tone at the two time-points studied, vessels were exposed to U-46619 (a thromboxane receptor agonist, 1 µM) for 20 min as a pre-constrictor. To directly assess the function of vascular smooth muscle cells, sodium nitroprusside (SNP, 50 µM) was applied after the final ACh concentration-response curve in the continued presence of the inhibitors. Finally, vessels were suffused with 0 mM Ca^2+^ buffer containing 2 mM EGTA and passive pressure-diameter relationships were determined across a pressure range of 5–50 mmHg. Changes in intraluminal diameter in response to alterations in intraluminal pressures or dilator agents were monitored using an inverted microscope (Olympus IX70, St Louis, United States) and a video-based caliper together with an A-D data processing system (ADInstruments, Colorado Springs, United States).

### Data handling and statistical analyses

Expression data for each vessel type were analyzed for age effects using one-way analysis of variance (ANOVA) together with Dunnett’s post-test. Day 3 was chosen to represent control. Comparisons between vessel types were performed using two-way ANOVA together with Bonferroni’s multiple comparison test (GraphPad Prism, CA, United States). Statistically significant differences were identified at probability values of *p* < 0.05. For functional studies all vessel diameters were normalized to the passive diameter at 30 mmHg (%D_30_passive).

## Results

### Developmental characteristics of mRNA expression for elastic fiber related genes in rat small cerebral and mesenteric arteries

**FIGURE 2:**
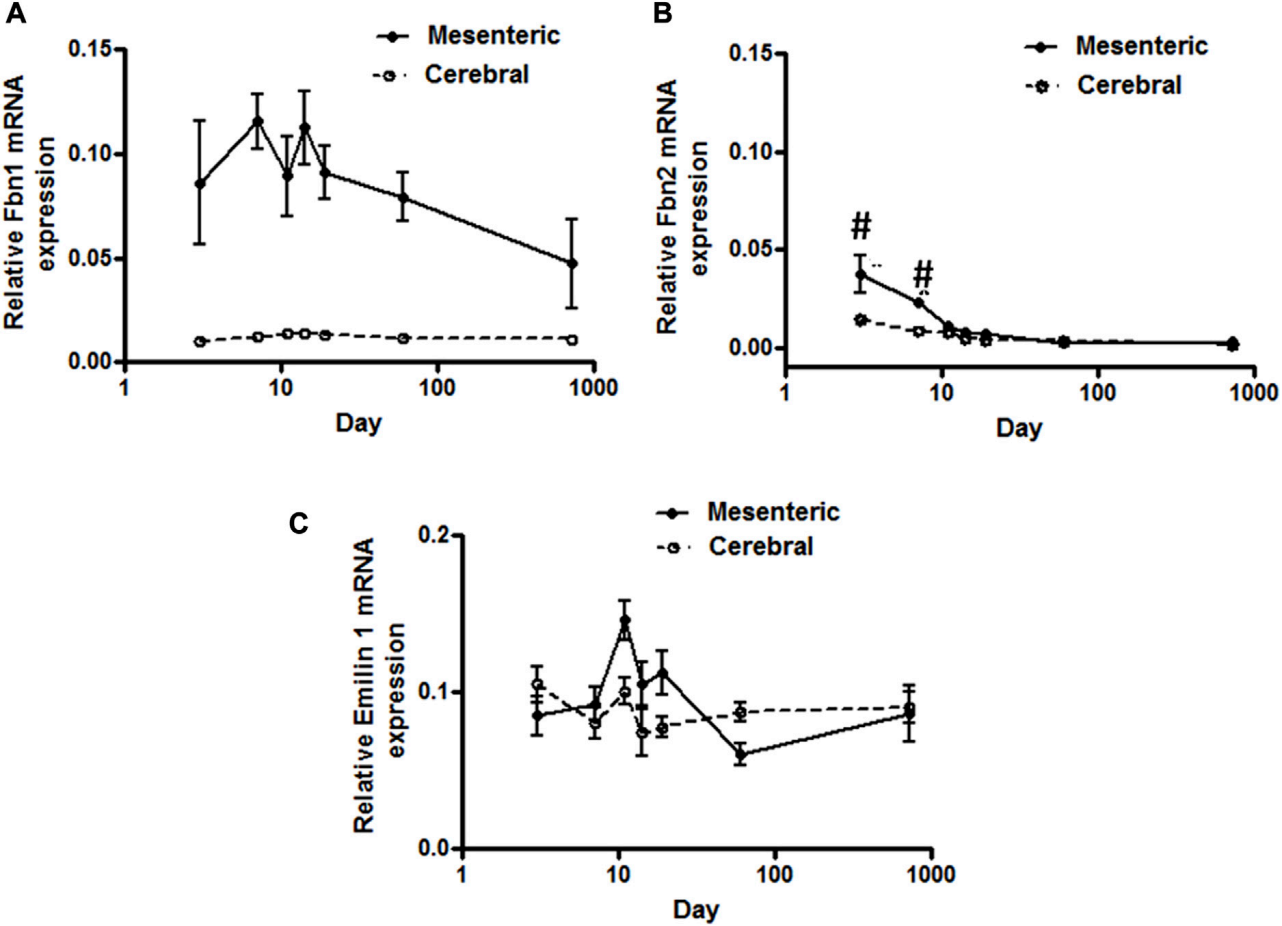
Elastic-fiber-associated microfibrils gene expression in rat cerebral and mesenteric arteries. Temporal relative mRNA expression profiles for **(A)** fibrillin-1 **(B)** fibrillin-2 and **(C)** Emillin-1 in rat day 3 through 2 years old. X axis (age) is shown as a log scale. Fibrillin-1 mRNA level in mesenteric artery showed higher levels of expression compared to cerebral arteries (two-way ANOVA) which remain almost constant throughout all ages. Fibrillin-2 mRNA expression levels in mesenteric and cerebral arteries showed distinct patterns at early postnatal day 3 and 7 but overlapping patterns in all later time periods (#*p* < 0.05 between vessel types). A very low level of mRNA expression for Emilin-1 was detected in both vasculatures.

Using a SYBER green-based approach, qPCR analysis demonstrated that cerebral artery elastin mRNA levels rise significantly from postnatal day 3 to a peak at 11–14 days after which expression appears to decline significantly throughout life (*p* < 0.05, one-way ANOVA, Figure 1A). Similarly, elastin mRNA expression for small mesenteric arteries peaked at postnatal day 11 (relative expression 22.4 ± 1.2) which was significantly greater than for cerebral vessels (relative expression 12.5 ± 1.1) (*p* < 0.05, two-way ANOVA). Although elastin mRNA levels for small mesenteric arteries declined throughout life, they remained elevated compared to the cerebral vessels (Figure 1A). Both the patterns of elastin mRNA expression and differences in magnitude of expression between vessel types were confirmed using a TaqMan assay system (see Supplementary Figure S2). As LOX is a key enzyme for the intramolecular cross-linking of both elastin fibers and collagen, thus contributing to the formation of elastin-rich sheet-like structure of the IEL (Rodriguez et al., 2008), its mRNA expression levels were similarly examined. LOX mRNA expression, in both vessel types, reached a peak around days 14–19 before declining between days 19 and 60 to a low level maintained through 2 years of age. During the peak expression period, LOX mRNA levels were greater in mesenteric vessels compared to cerebral arteries (Figure 1B). In addition to LOX, mRNA expression levels for t-TG2 were also examined because of its previously reported crucial role in the formation of the beaded outer microfibrils for construction of mature elastic fibers (Kielty et al., 2002). As shown in Figure 1C, the mRNA expression patterns for transglutaminase 2(t-TG2) were markedly different compared to that for elastin and LOX. t-TG2 mRNA rose slightly and gradually from postnatal day 3 through life in both vessel types with a significant increase at 2 years compared to control (3 day, *p* < 0.05 one-way ANOVA. t-TG2 mRNA levels were also significantly greater in mesenteric compared with cerebral arteries from day 11 through all later time points (*p* < 0.05, two-way ANOVA). Fibrillin-1 & 2, mRNA expression was similarly examined because of their reported role as major structural elements of fibrillin-rich microfibrils in ECM assemblies (Kielty et al., 2002; Wagenseil and Mecham, 2007). Although expressed at very low levels, fibrillin-1 mRNA in mesenteric arteries was higher compared to cerebral arteries in which expression levels were largely unchanged throughout life (Figure 2A). At days 3 and 7 fibrillin-2 mRNA levels were significantly (*p* < 0.05, two-way ANOVA) greater in mesenteric arteries compared to cerebral vessels while at later time-points expression was similar (Figure 2B). mRNA expression of Emilin1 (“elastin microfibril interface located protein”) showed a similar multiphasic expression pattern in and mesenteric arteries while remaining relatively constant in cerebral vessels (Figure 2C).

### Developmental characteristics of mRNA expression for collagen family genes in rat cerebral and small mesenteric arteries

**FIGURE 3:**
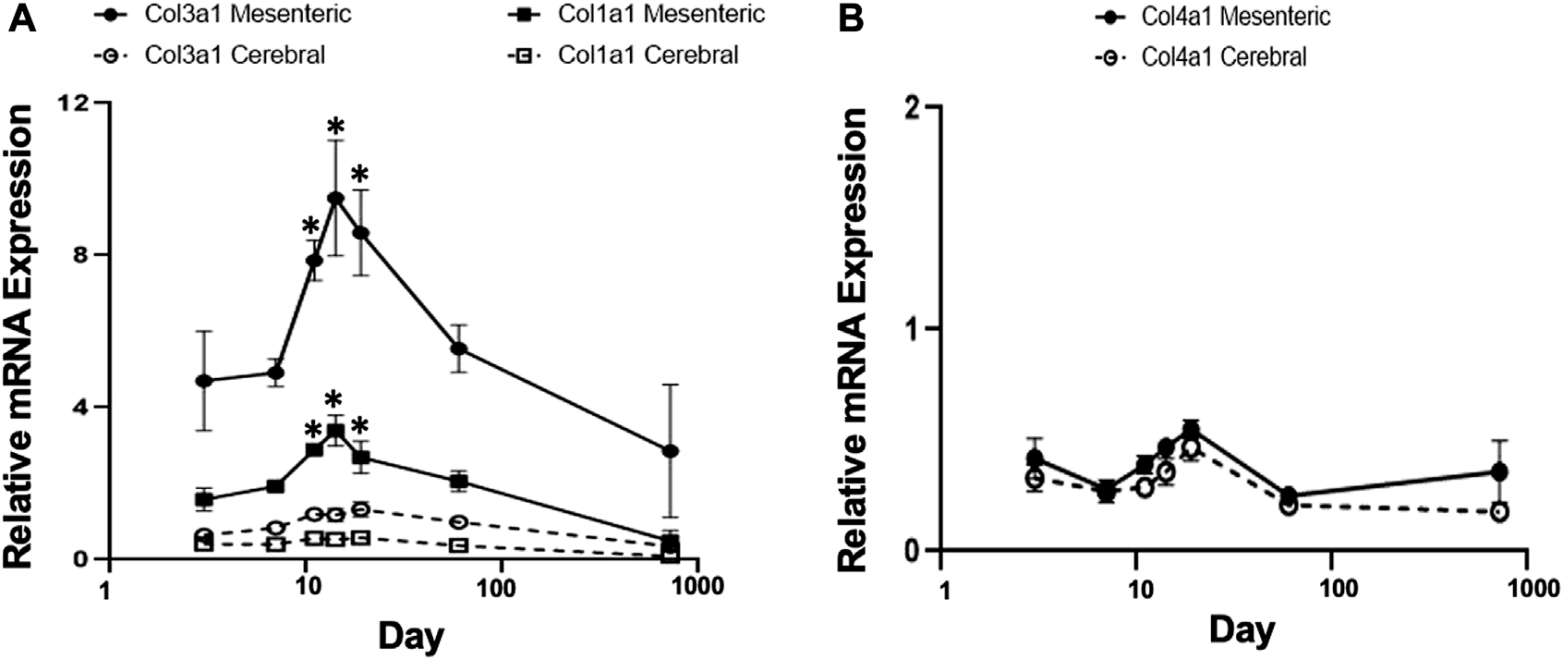
Gene expression profiles of fibrillar collagen family in rat cerebral vs. mesenteric arteries. Age-related profiles of gene expression of **(A)** fibrillar collagen types I, and III, and **(B)** collagen type IV in rat resistance arteries performed by TaqMan assay. Collagen types I and III, showed a similar mRNA expression pattern compared to elastin but at lower absolute levels. Collagen type 3 mRNA expression was significantly higher in mesenteric vessels compared to cerebral arteries at all time points (two-way ANOVA) and was expressed at higher levels than collagen type 1 mRNA. A relatively constant level of collagen type IV mRNA expression was detected in both vessel types. X axis (age) is shown as a log scale. β-actin gene expression used as reference gene and changes in mRNA expression was calculated as fold changes relative to elastin expression of calibrator, a 2 month old cerebral artery. Results are shown for n = 4–5 separate experiments and are presented as mean ± SEM. *p < 0.05 compared to day 3 for respective vessel type; one way ANOVA.

TaqMan assays were used to quantify mRNA levels for collagen types I, II, III, and IV and to determine developmental differences in expression patterns between mesenteric and cerebral arteries. Fibrillar types I and III collagens showed mRNA expression patterns similar to that of elastin although at lower absolute levels (Figure 3A compared to Figure 1A). Thus, mRNA for both proteins peaked at approximately 11–14 days with significantly higher levels being apparent in mesenteric vessels compared to cerebral arteries (*p* < 0.001, two-way ANOVA). In contrast, expression of collagen Type II mRNA was not detectable in either vessel type while mRNA for basement membrane type IV collagen was relatively constant throughout life and of similar magnitude in both vessels (Figure 3B).

### Elastin deposition in the intact artery wall

**FIGURE 4:**
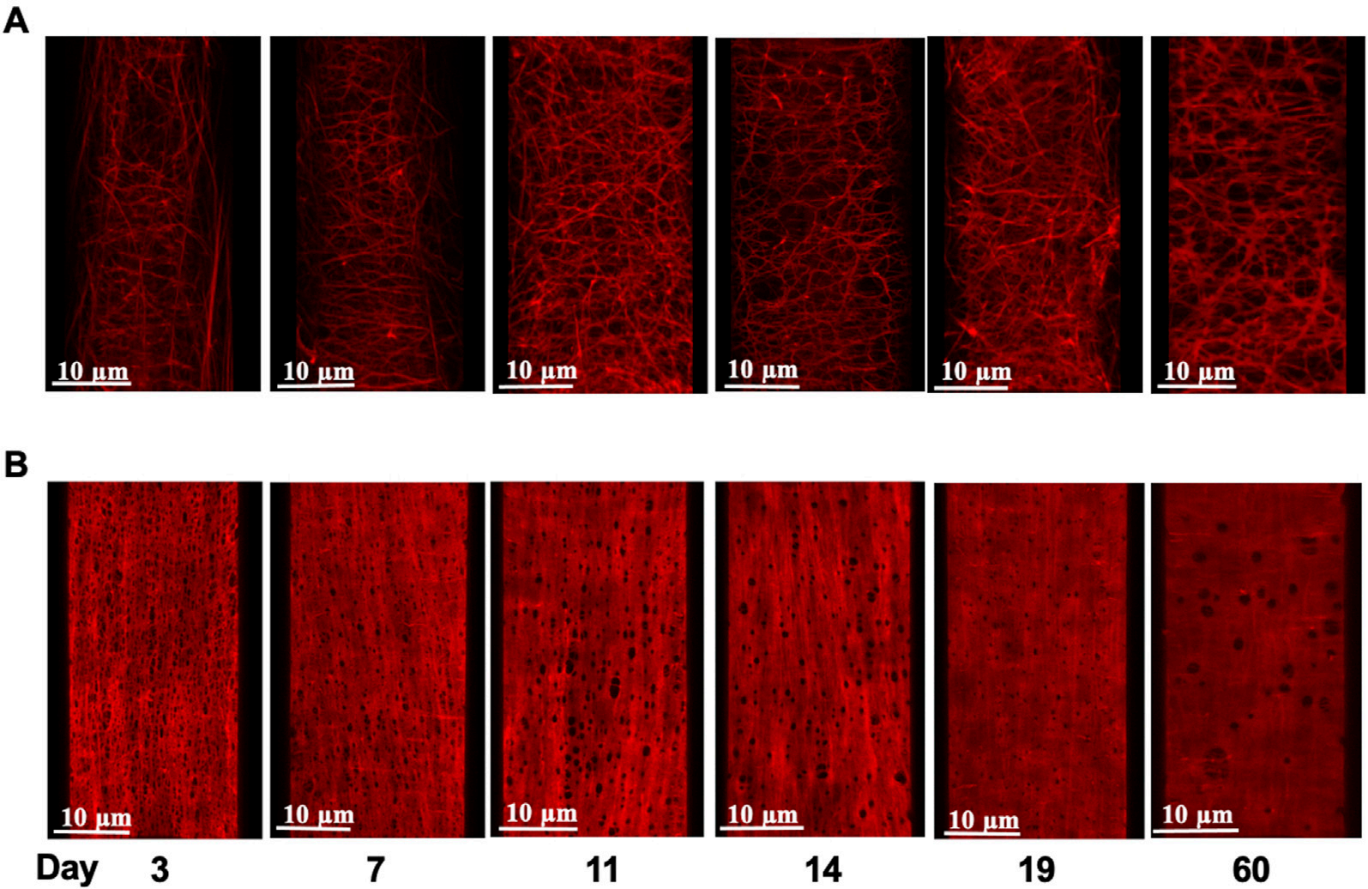
Age-dependent elastin deposition in the rat mesenteric artery wall. Example images of Alexa 633 hydrazide stained adventitial fibers **(A)** and internal elastic lamina [**(B)**; IEL] in mesenteric arteries from rats aged 3–60 days (left to right). Images shown in panels A and B are from the same artery at each age.

**FIGURE 5:**
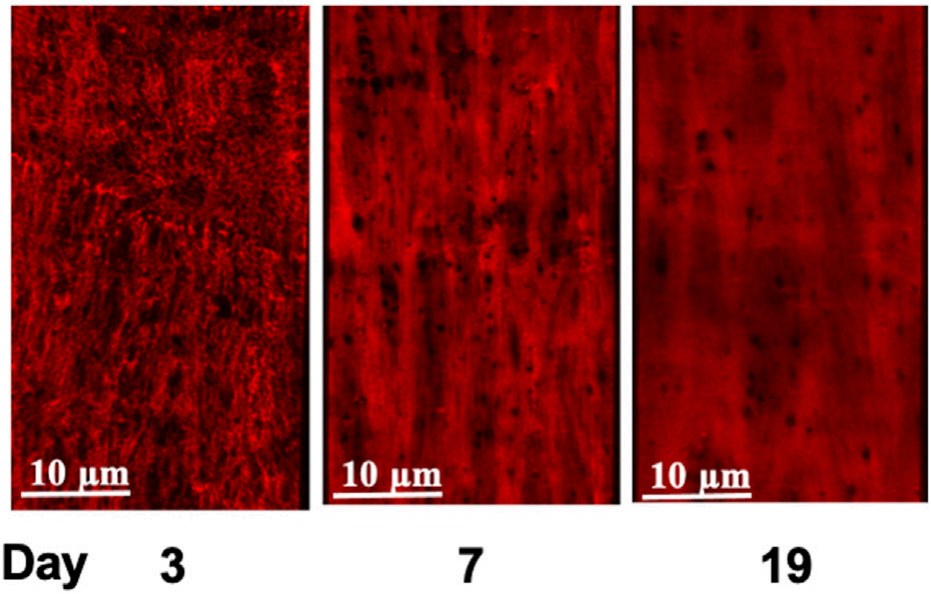
Age-dependent elastin deposition in the rat cerebral artery wall. Alexa 633 Hydrazide staining of the Internal Elastic Lamina, (IEL, red) of cerebral arteries at postnatal ages of 3, 7, and 19 days. Consistent with earlier studies (Clifford et al., 2011; Hill et al., 2016), cerebral arteries did not show elastin staining in the adventitia. An equivalent set of images incorporating Yo-Pro-1 iodide staining of cell nuclei to show orientation of the cellular components (smooth muscle cells circumferential and endothelial cells longitudinal) is provided in Supplementary Figure S4.

As we reported in earlier studies (Clifford et al., 2011), elastin fibers in the walls of small arteries from adult rats (2 months) exhibit an intricate 3D arrangement within the vascular wall and show marked differences between various small arteries in elastin content and spatial distribution. To extend this study, 3D confocal microscopy was performed on cannulated pressurized cerebral and mesenteric arteries from all age groups described above to examine developmental changes in the expression and distribution of small artery wall matrix proteins. Z-stack images from cerebral arteries at postnatal ages of 3, 7, and 19 day-old (Figures 4A,B, respectively) show the absence of adventitial elastin layer in these vessels confirming our earlier finding and showing that this occurs throughout life. A progressive development of cerebral artery IEL was also apparent across postnatal ages of 3, 7, 11, 14, 19 days and 2 months. This was evident as a progression from a fibrous sheet like appearance towards more well-defined and solid appearing sheet with fenestrae (Figure 4, left to right, respectively). Similarly, the IEL of small mesenteric arteries showed a postnatal development towards a more uniformly fenestrated sheet (Figure 4, bottom images). The adventitial elastin component of mesenteric arteries appeared to become denser with age (Figure 5; top images (A) 3 day, (B) 14 day, and (C) 19 day. Z-stack images at the level of the media showed accumulation of higher elastin content at the adventitial layers of mesenteric arteries from 3, 7, 11, 14, 19 day-old and 2 months rats (Figure 4A from left to right, younger to older accordingly). This occurred in parallel with the development of IEL from a fibrous appearing form into a continuous fenestrated sheeted from (Figure 4B; Figure 5).

**FIGURE 6:**
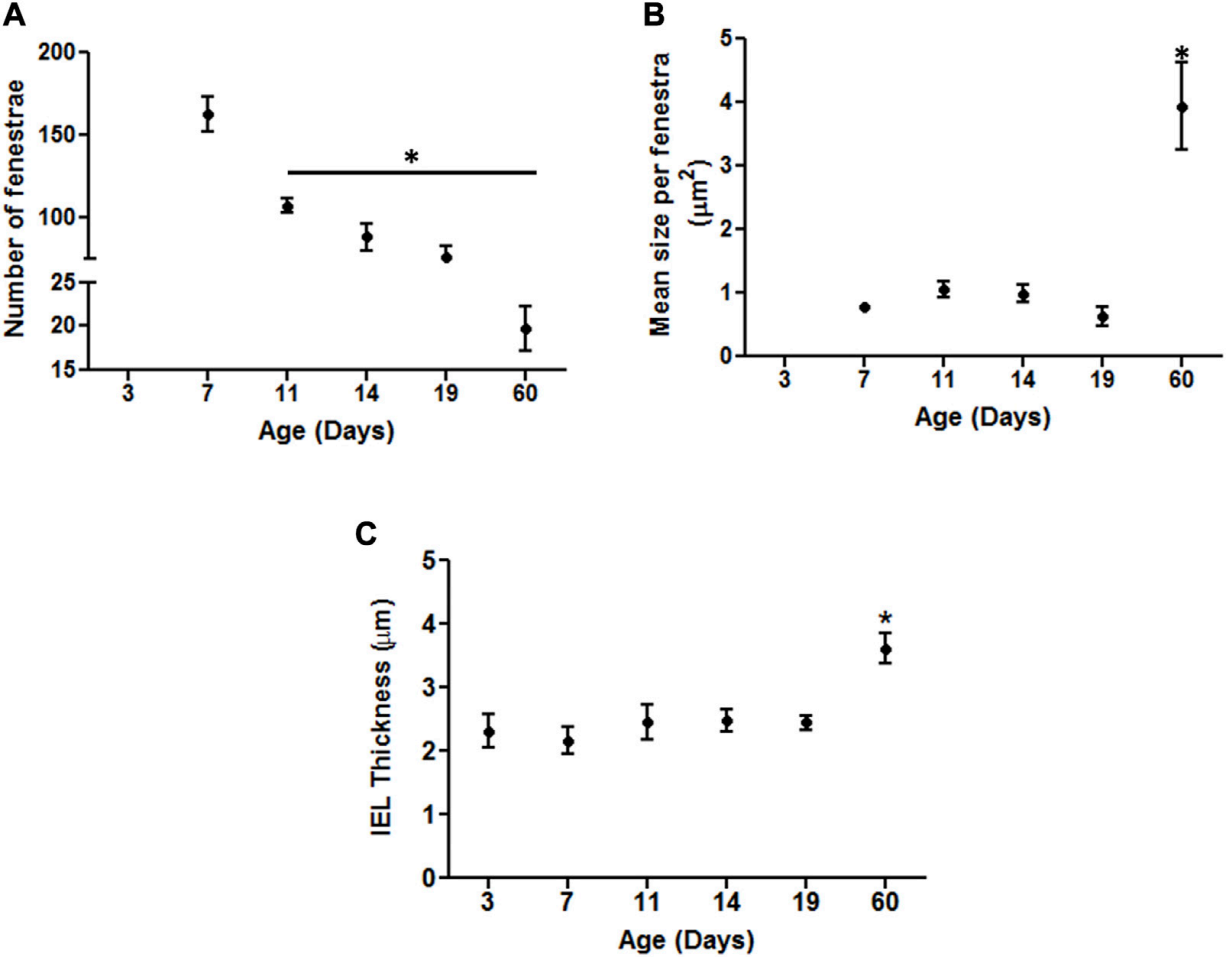
Developmental changes in the characteristics of the internal elastic lamina of rat mesenteric small arteries. Quantitative comparison of IEL characteristics in mesenteric arteries relative to age as demonstrated by elastin staining. **(A)** Numbers of fenestrae within representative area of 60 × 120 µm in rat mesenteric small arteries revealed continuous decrease with the maximum at 7 day vs. the minimum at 60 day -old vessels. **(B–C)**: The mean size per fenestra and IEL thickness within representative area of 60 × 120 µm represent the highest in older adult ages vs. the younger ones in rat mesenteric arteries. Results were expressed as the mean ± SEM of n = 4–5 separate image analysis per vasculature/age. Note, fenestrae were not measured at day 3 due to the loose mesh appearance of the IEL. * *p* <0.05 compared to day 3.

In an attempt to provide a quantitative analysis for elastin expression at the protein level, we determined fluorescence intensity (of Alexa Fluor 633 hydrazide) within regions of interest (ROI) while doing this at defined locations within the z-stack set of images. As shown in Figure 1A, throughout life, total elastin content of cerebral arteries was significantly lower than for mesenteric vessels. This is consistent with cerebral vessels lacking adventitial elastin. As shown in Figure 4B, the content of elastin in the IEL was relatively constant throughout postnatal life whereas total expression increased supporting the suggestion of elastin accumulation in the adventitia in older ages. The numbers of fenestrae (within a ROI of 60 × 120 µm) in rat mesenteric small arteries decreased from an apparent maximum at day 7 to a minimum at 2 years of age (Figure 6A). Note that we were not able to conduct this measurement at 3 day-old mesenteric arteries because of open mesh network characteristic of fibers within the IEL. Thus, identification and definition of clearly distinguishable fenestrae was not possible. The mean size of mesenteric fenestrae and IEL thickness increased significantly but only after the postnatal period (Figures 6B,C). Collectively these studies indicate that there is postnatal development of elastin networks in the walls of small arteries which raises questions as to the functional significance of these anatomical changes.

### Developmental changes on ACh-induced relaxation of mesenteric and cerebral arteries of postnatal 3 and 19 day-old rats

**FIGURE 7:**
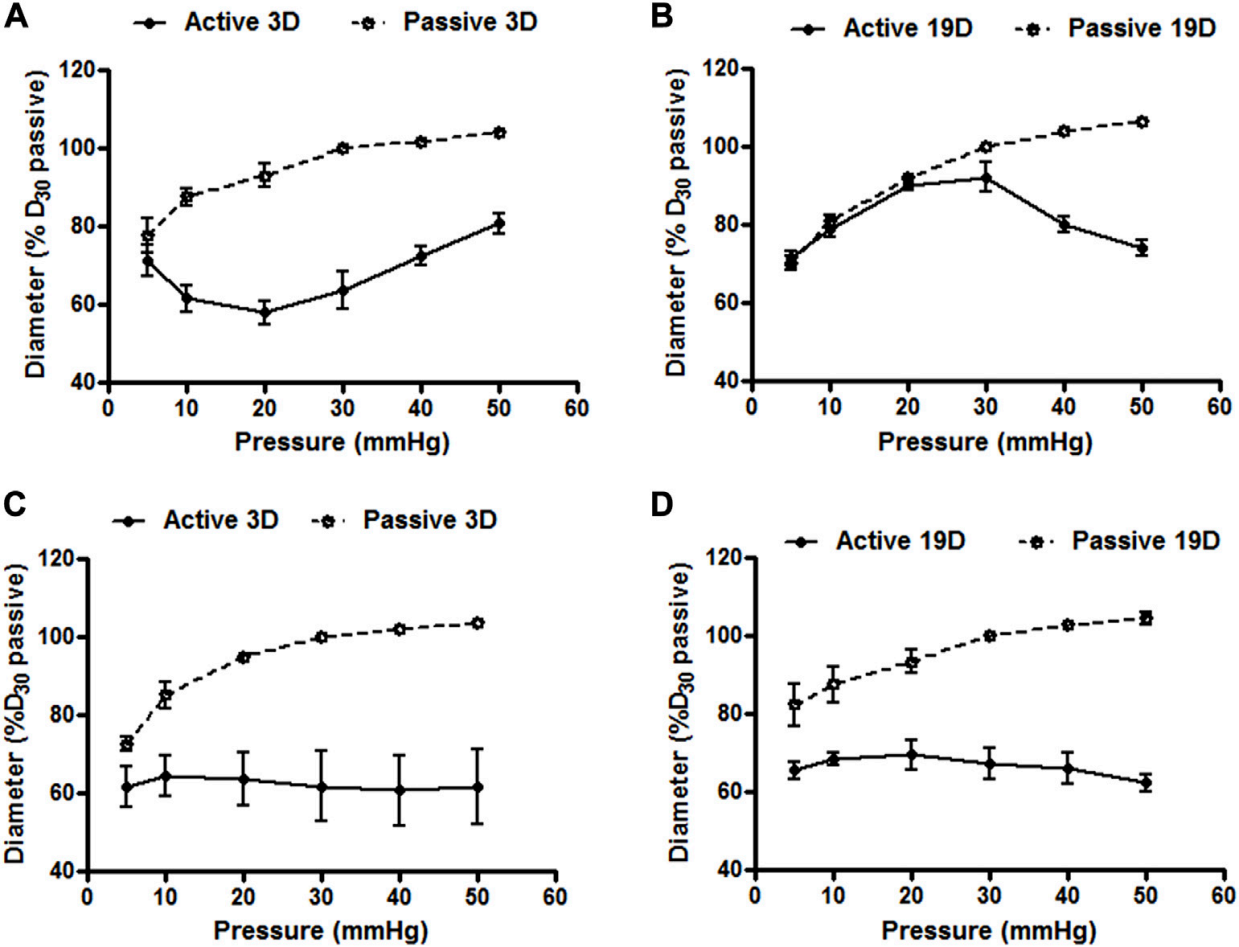
Cerebral and mesenteric artery myogenic responses at postnatal days 3 and 19. Myogenic responses induced by varying intraluminal pressure, *in vitro*, in rat cerebral arteries at postnatal day 3 **(A)** and 19 **(B)** day-old rats showed distinct patterns. Myogenic constriction of day 3 cerebral arteries was observed at relatively lower intraluminal pressure (5–20 mmHg) vs. day 19 which showed myogenic constriction at pressure >30 mmHg. Pressure induced vasoconstriction was nearly identical in arteries from rats at day 3 **(C)**, and 19 **(D)**. Results are expressed as the mean ± SEM of n = 5 separate experiments.

To determine whether small artery function showed developmental changes in the postnatal period we compared vasomotor responses of cannulated mesenteric and cerebral arteries at postnatal days 3 and 19. Emphasis was placed on myogenic responsiveness and endothelial-dependent vasodilation. As shown in Figure 7, although differences in patterns were apparent, both the cerebral and mesenteric arteries showed myogenic responsiveness at, postnatal day 3 and 19 consistent with development of a level of vascular smooth muscle cell-mediated contractile activity. Of potential significance was the observation that myogenic constriction of day 3 cerebral arteries were left-shifted on the pressure scale and occurred at lower intraluminal pressures (5–20 mmHg; Figure 7A) compared to day 19 at which time the myogenic response curve was more right-shifted with myogenic constriction expressed at intraluminal pressures over 30 mmHg (Figure 7B). Pressure-induced vasoconstriction was, however, nearly identical between day 3 and 19 mesenteric arteries (Figures 7C,D). Thus, these data suggest that mechanotransduction processes underlying myogenic function of mesenteric and cerebral arteriolar VSMCs may be fully developed in the immediate postnatal period. However, full expression of the vascular myogenic response develops in cerebral vessels as aging occurs shifting right ward on the pressure scale, perhaps paralleling and adapting to developmental increases in systemic pressure.

**FIGURE 8:**
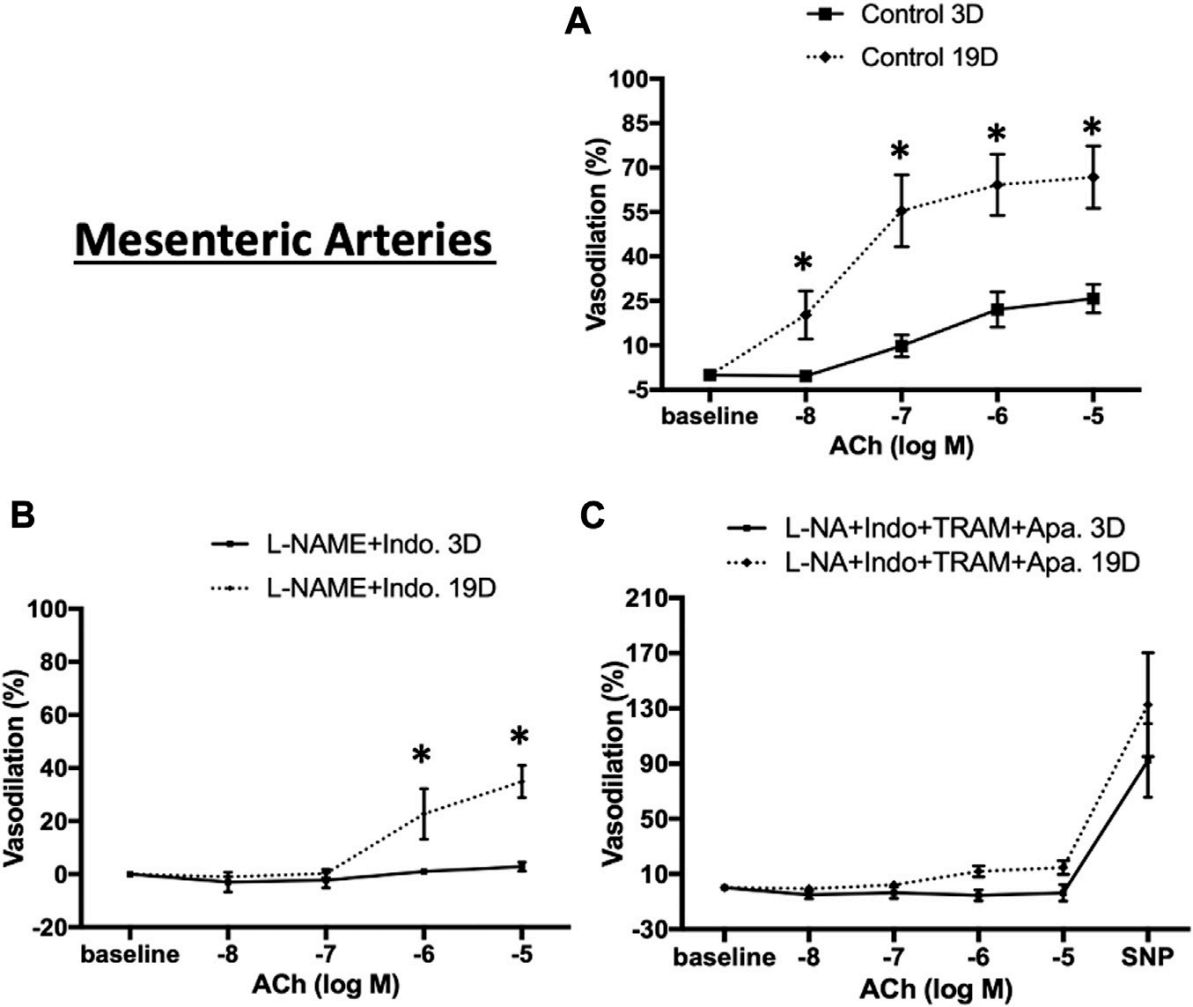
ACh-induced relaxation of 3 and 19 day-old rat mesenteric small arteries. ACh-induced relaxation in mesenteric arteries from 3 to 19 day-old rats in **(A)** control (no treatment) **(B)** treatment with L-NAME plus indomethacin, and **(C)** combination of L-NAME, indomethacin, TRAM-34, and apamin. ACh-induced vasodilation in 3 day-old mesenteric arteries was markedly lower than 19 day-old mesenteric arteries which almost completely abolished in day 3 vessels after treatment with L-NAME and indomethacin. Endothelium-dependent vasodilation was significantly diminished after combination treatments by all inhibitors in both ages. Results were expressed as the mean ± SEM of n = 4 separate experiments. Maximum diameter was determined following superfusion of vessels with Ca^2+^ free buffer containing 1 mM EDTA. * *p* <0.05 between groups.

To test whether endothelial cell (EC) function changes during the postnatal period, ACh-concentration-response curves were examined in cerebral and mesenteric arteries at postnatal days 3 and 19. Experiments were performed in the absence or presence of L-NAME (NOS inhibitor), indomethacin (prostaglandin synthase inhibitor), apamin (SK_Ca_ blocker), and TRAM-34 (IK_Ca_ blocker) to examine mechanisms contributing to EC-dependent vasodilation. ACh-induced vasodilation in 3 day-old mesenteric arteries was markedly attenuated compared to day 19 arteries (Figure 8A, maximal relaxation: 25.8% ± 4.8% vs. 66.8% ± 10.5% respectively) suggesting developmental changes in EC-mediated vasodilation during the postnatal period. After blockade of two major paracrine vasodilators (NO and PGI_2_), ACh-mediated vasodilation in 19 day-old mesenteric arteries reduced significantly to a maximal dilation of 34.9% ± 6.1% vs. control whereas EC-dependent vasodilation was almost completely abolished in 3 day-old vessels (Figure 8B). To examine any contribution of hyperpolarization mediated by small (SK_Ca_) and intermediate (IK_Ca_) conductance Ca^2+^-activated K^+^ channels, the 3 and 19 day-old mesenteric vessels were also treated with a combination of L-NAME, indomethacin, TRAM-34 (IK_Ca_ blocker), and apamin (SK_Ca_ blocker). The combination of all four inhibitors caused complete inhibition of ACh-induced vasodilation in day 19 mesenteric arteries (Figure 8C) suggesting that a hyperpolarization component of EC-dependent dilation develops during the postnatal period.

**FIGURE 9:**
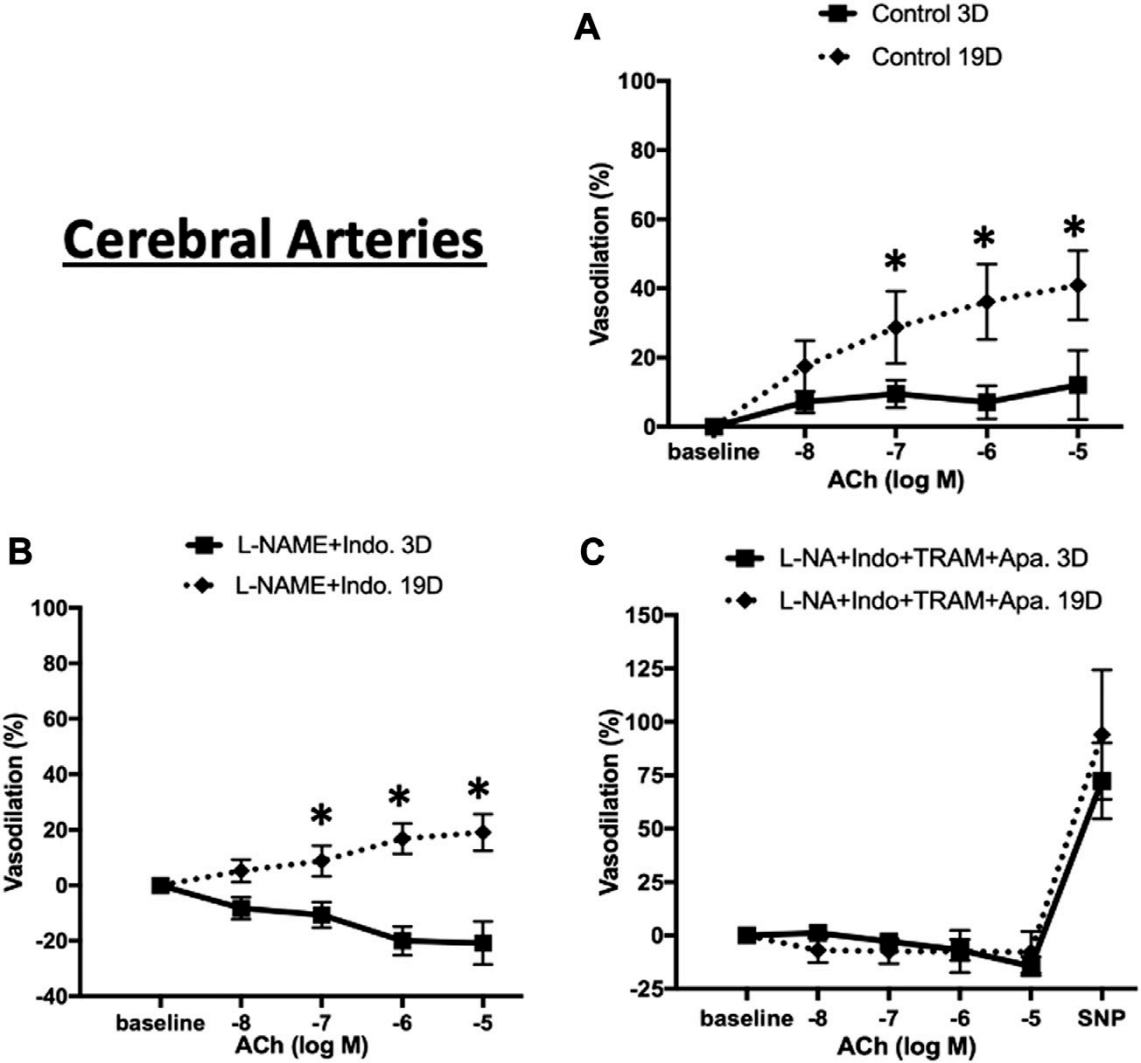
ACh-induced relaxation of 3 and 19 day-old cerebral arteries. ACh-induced relaxation in cerebral arteries from 3 to 19 day-old rats in **(A)** control (no treatment) **(B)** treatment with L-NAME plus indomethacin, and **(C)** combination of L-NAME, indomethacin, TRAM-34, and apamin. At day 3 ACh dilated cerebral to a lesser extent than at day 19 with the response abolished by treatment with L-NAME and indomethacin. Endothelium-dependent vasodilation was abolished after combination treatments by all inhibitors at both ages. Results were expressed as the mean ± SEM of n = 5–6 separate experiments. * *p* <0.05 between groups.

We similarly expanded the above studies of EC-dependent vasodilator function to cerebral arteries at postnatal days 3 and 19. As shown in Figure 9A, in the absence of inhibitors ACh-induced relaxation was evident at both ages but at a considerably lower magnitude in the younger animals (maximal relaxation 40.9% ± 10.0% in 19 day vs. 12.1% ± 9.9% in 3 day-old cerebral vessels. The combination of L-NAME and indomethacin totally abolished Ach-induced dilation 3 day-old cerebral arteries while at day 19 the vessels dilated to 19.1% ± 6.6% (Figure 9B). Consistent with the mesenteric data, at day 19, ACh-induced vasodilation was abolished in the presence of the four inhibitors (Figure 9C).

To determine whether the age-dependent differences in vasodilation reflected a smooth muscle component, responsiveness to the NO donor, SNP (50 µM), was examined. Robust and equivalent dilator responses were observed to SNP in both vascular beds and at both time-points (Figures 8C, 9C). Collectively, the data suggest an age-dependent maturation in an EDH-dependent component of vasodilation.

### mRNA expression profiles for endothelium-dependent smooth muscle cell vasodilation factors in cerebral and mesenteric arteries from 3 to 19 day-old rats

While argument could be made that the differences in the functional data indicate maturation of myoendothelial signaling, including development of the IEL, alterations in the expression of various ion channels and enzymes could also contribute. As such, we performed additional qPCR experiments on cerebral and mesenteric arteries to determine the relative mRNA expression levels of endothelial nitric oxide synthase (eNOS), prostaglandin-endoperoxide synthase 2 (Ptgs2), endothelial small (SK_Ca_), intermediate (IK_Ca_), and large (BK_Ca_) conductance Ca^2+^-activated K^+^ channels at postnatal days 3 and 19. In cerebral arteries, SK_Ca_, eNOS3, and α-BK_Ca_ mRNA levels were significantly increased at day 19 compared to the day 3 groups (Supplementary Figure S3A). No significant change in mRNA expression was detected for Ptgs2 while a small, but significant, decrease in IK_Ca_ mRNA expression was detected at day 19 compared to the day 3 (Supplementary Figure S3A). As shown in Supplementary Figure S3B, qPCR analysis for mesenteric arteries demonstrated that the abundance of eNOS3 and IK_Ca_ mRNA was similar in both age groups studied while there were significant increases in levels of mRNA expression for α-BK_Ca_, SK_Ca_, and Ptgs2 at day 19 compared to day 3. A comparison between cerebral and mesenteric expression levels is shown in Supplementary Figure S3C.

As development could conceivably alter the expression of the house keeping genes used for normalization, we examined both β-actin and GAPDH expression levels. In these experiments β-actin and GAPDH show consistent levels of expression at days 3 and 19 for both vasculatures (Supplementary Figure S1). Parallel qPCR analyses using GAPDH, confirmed gene expression levels with the exception of an age-dependent difference in expression for IK_Ca_ in cerebral vessels.

## Discussion

In addition to the ECM (adventitial, medial and internal elastic lamina components) impacting the mechanical properties of blood vessels, the ECM is known to affect cell signaling in the resistance vasculature through interactions with cell surface receptors including integrins (Sun et al., 2008; Sun and Meininger, 2011). Further, the fenestra in the internal elastic lamina provide conduits for formation of the myoendothelial junction that underlie vasomotor responses mediated by hyperpolarization (Halper and Kjaer, 2014). Thus, the contribution of the ECM to both the mechanical and functional properties of the vascular wall in normal and diseased states (Baumbach et al., 1988; Hill and Ege, 1994) underscores the importance of developing a deeper understanding of the ECM in resistance arteries.

The results of the current studies show that during the postnatal period, small arteries of the rat continue to undergo molecular and structural remodeling. Between day 3 and 19 of life, expression levels of a number of ECM proteins (at the mRNA level) exhibit a distinct peak, while elastin continues to be deposited in the adventitia and IEL holes appear to mature. Coincident with these changes in structure, aspects of functional control continue to develop. Specifically, while nitric oxide-mediated pathways of vasodilation were apparent at the earliest time-point examined (day 3) EDH-dependent mechanisms were not apparent at day 3 but were evident by day 19. Further, myogenic responsiveness of cerebral vessels showed a shift to higher intraluminal pressure between postnatal days 3 and 19. Although difficult to demonstrate a direct causal relationship between the structural and functional events at this time, small arteries clearly continue to develop in the immediate postnatal period.

Elastin expression of large vessels (such as mouse aorta) is greatest during the last third of embryonic development, beginning around embryonic day 14 through postnatal days 7–10 (Mecham, 2008; Wagenseil et al., 2010). In adulthood there is a relative inability to synthesize and assemble mature elastic fibers, a phenomenon which is thought to contribute to age-related changes in tissue stiffness (Parks, 1997; Vrhovski and Weiss, 1998) as well as in pathological states including supravalvular aortic stenosis, aneurysm and hypertension (Martinez-Revelles et al., 2017; Adeva-Andany et al., 2021; Brengle et al., 2023). In regard to elastin in small arteries, the present studies showed that tropoelastin mRNA expression exhibits a distinct postnatal peak, initially increasing at days 3–5 and reaching a maximum at days 9–11. Thereafter, tropoelastin mRNA expression decreased through progression to adulthood. A similar pattern was observed in proteins important to mature elastogenesis including the cross-linking enzyme, lysyl oxidase, the microfibrillar proteins fibrillin-1,2 and emilin-1. Consistent with our earlier findings of differing elastin content in mesenteric and cerebral vessels (Clifford et al., 2011), tropoelastin and lysyl oxidase mRNA expression was higher in mesenteric vessels compared to that in cerebral arteries, although similar temporal changes were observed in both vascular beds. The finding of an early postnatal peak in elastin expression is also consistent with that reported for aorta in earlier studies (Wagenseil et al., 2010). Factors driving this postnatal increase in elastin expression are not fully understood although cardiac output and arterial pressure also increase in the rat during this period (Litchfield, 1958) perhaps providing a mechanical stimulus. Humans, in contrast, show higher systemic blood pressures at birth. A postnatal increase in mRNA expression was also observed for the type 1 and 3 fibrillar collagens in both mesenteric and cerebral vessels. The temporal pattern of expression for these collagen species was again similar to that for elastin – peaking at approximately days 9 – 11 thereafter declining as the rats progressed to adulthood. Also similar to elastin, fibrillar collagen expression was greater in mesenteric vessels as compared to small cerebral arteries. In contrast to the fibrillar collagens basement membrane type IV collagen, which assembles as a sheet-like structure, did not change postnatally and expression levels were similar in mesenteric and cerebral vessels at all ages studied.

Using 3D confocal microscopy and elastin staining using Alexa 633 hydrazide, together with image reconstruction it was evident that elastin rich components of the vessel underwent a postnatal phase of structural organization. In particular, the IEL showed an apparent maturation of fenestrae characterized by a marked decrease in number from postnatal day 3 to day 60. Hole size was relatively constant in the early post-natal period but significantly increased by day 60.

A number of studies have proposed that IEL holes allow for sites of communication between VSM and ECs via myoendothelial junctions (Sandow and Hill, 2000; Sandow et al., 2002). As these junctions facilitate endothelial-dependent hyperpolarization and vasodilation we hypothesized that vessel function would develop along with the changes in IEL structure. To examine vasomotor function mesenteric and cerebral vessels were isolated and cannulated for pressure myography at days 3 and 19. Myogenic tone and pressure-induced constriction were evident at day 3, in both vessel types, consistent with functional vascular smooth muscle. Interestingly while the myogenic responsiveness of mesenteric vessels was similar at day 3 and day 19, in cerebral vessels the pressure diameter relationship shifted to the right (i.e. to higher pressures) with age. This may reflect the well-described ability of cerebral vessels to autoregulate around their mean intraluminal pressure and that mean systemic pressure in a day 3 rat has been reported to be approximately 22 increasing to 86 mmHg by day19 (Litchfield, 1958).

To examine vasodilator responsiveness the cannulated small arteries were treated with ACh. While ACh responsiveness was evident in both vessel types at day 3 there was a significantly increase in magnitude by day 19. Treatment of vessels at day 3 with a combination of L-NAME and indomethacin completely abrogated dilator responses ACh suggesting at this age the vasodilation resulted from paracrine production of NO and/or prostaglandins. In contrast at day 19 a component of ACh-induced dilation persisted in the presence of L-NAME and indomethacin and was blocked by the K^+^ channel blockers, TRAM and apamin. These data suggest that an EDH-dependent component of dilation develops postnatally in small cerebral and mesenteric arteries and does so in parallel with apparent structural maturation of the IEL. In earlier studies, Sandow et al. also reported developmental changes in the relationship between EDH and maturation of the IEL with saphenous arteries of juvenile rats (2 weeks of age) demonstrating EDH-dependent relaxation which was then lost in the young adult (12 week) (Sandow et al., 2004). The loss of EDH-dependent relaxation was associated with an age-dependent decline in numbers of MEGJs although IEL number was abundant at both ages studied. At this point, however, it is not possible for the current study to ascribe a direct causal relationship as other components of the vascular wall, and within MEJs, may well be developing. Indeed, we observed that mRNA expression for key K^+^ ion channels (SKCa, IKCa and BKCa) and enzymes (NOS and PG synthase) may be changing during this period of development in the rat (Supplementary Figure S2). Clearly these events, particularly as relates to SKCa and IKCa may also impact the final maturation of functional MEJs. Similarly, the significance of the marked increase in BKCa expression between postnatal days 3 and 19 requires further study, as BKCa is known to be a significant regulator of smooth muscle vasomotor tone and, in particular, myogenic reactivity of small arteries (Schubert and Nelson, 2001; Yang et al., 2013).

Interestingly, while the EDH-dependent component of ACh-induced dilation developed between postnatal days 3 and 19, robust responsiveness to sodium nitroprusside was evident at both time points. Consistent with the myogenic responsiveness data this suggests that smooth muscle vasomotor function is well developed in the rat by day three of life.

It should be noted that the present study has a number of limitations which should be addressed in future studies. Firstly, we cannot definitively link the postnatal changes in elastin expression and organization with the parallel changes in vasomotor responsiveness. Secondly, we did not take an unbiased approach to selecting ECM targets although the approach was supported by previous studies of large vessels and our own studies showing differences in elastin content between cerebral and peripheral small arteries (Clifford et al., 2011; Hill et al., 2016). Further, the use of whole vessel homogenates in the PCR studies conceivably reflects multiple cell types although a similar limitation would likely occur in studies using a more unbiased target approach. As a result, our studies do not consider the cellular source of the matrix proteins studied (Lin et al., 2019) and they focus on two vascular beds although considerable heterogeneity is known to occur throughout the vasculature (Hill et al., 2016). Finally, future studies should take a systematic approach to evaluating the role of sex in the developmental changes in the ECM and their impact on function. Despite these limitations the present study demonstrates that small artery structural remodeling and aspects of functional vasomotor control continue to develop in the immediate postnatal period in the rat. Clearly the parallel correlation of ECM expression and control indicates the causative links need to be further explored.

## Data Availability

The raw data supporting the conclusion of this article will be made available by the authors, without undue reservation.
