# Supplementary material for: Postnatal development of extracellular matrix and vascular function in small arteries of the rat
Source: Front Pharmacol. 2023 Aug 15;14:1210128. doi: 10.3389/fphar.2023.1210128 (PMC10464837; doi:10.3389/fphar.2023.1210128)
Supplement: Supplementary file 1 [file DataSheet2.PDF]

SUPPLEMENTARY FIGURE LEGENDS:

Supplementary Figure 1: Steady level of β-actin and GAPDH expression through postnatal life of 3 and 19 day-old in rat cerebral and mesenteric arteries. A: β-Actin, and B: GAPDH used as housekeeping genes in qPCR performed on 3 and 19 day-old cerebral and mesenteric arteries. No significant difference between Ct values in qPCR analysis confirming that these housekeeping genes are not age-dependent.

Supplementary Figure 2: Confirmation of developmental changes in elastin mRNA expression in rat cerebral compared to mesenteric arteries. Temporal relative mRNA expression profiles for elastin in rat postnatal 3, 7, 11, 14, and 19 day, 2 month and 2 year old. X axis (age) is shown as a log scale. Data were obtained using TaqMan assay. GAPDH gene expression used as reference gene and changes in mRNA expression was calculated as fold changes relative to elastin expression of calibrator, an arbitrary 2 month old cerebral artery sample. Results are shown for n = 4-5 separate experiments and are presented as mean ± SEM. Similarly to the data shown in Figure 1A, elastin mRNA levels rise significantly from postnatal day 3 to a peak at 11 day after which expression also significantly declines throughout life in both vessel types.

Supplementary Figure 3: The α-BK_{Ca} mRNA expression increased dramatically in cerebral and mesenteric arteries of 19 day-old rats than 3 day. The fold relative mRNA expression profile of SK_{Ca}, eNOS3, Ptgs2, α-BK_{Ca}, and IK_{Ca} of A: cerebral and B: mesenteric small arteries in 3 vs 19 day-old rats. For both vasculatures significant increase in α-BK_{Ca} and SK_{Ca} mRNA expression levels were determined in 19 vs 3 day-old vessels followed by eNOS3 in cerebral and

Ptgs2 in mesenteric arteries. GAPDH gene expression used as reference gene. Changes in mRNA expression levels for each desired gene in day 19 were calculated separately as fold changes relative to expression of 3 day old samples as calibrator. Results are shown for n = 4-5 separate experiments run in triplicate and are presented as mean ± SEM.

Supplementary Figure 4: Example image usingYo-Pro-1 iodide staining of nuclei (green) showing the orientation of smooth muscle (circumferential) and endothelial (longitudinal) cells. Image is of vessels at days 3, 7 and 19 (left to right).
