# Supplementary figures and images for: Postnatal development of extracellular matrix and vascular function in small arteries of the rat
Source: Front Pharmacol. 2023 Aug 15;14:1210128. doi: 10.3389/fphar.2023.1210128 (PMC10464837; doi:10.3389/fphar.2023.1210128)

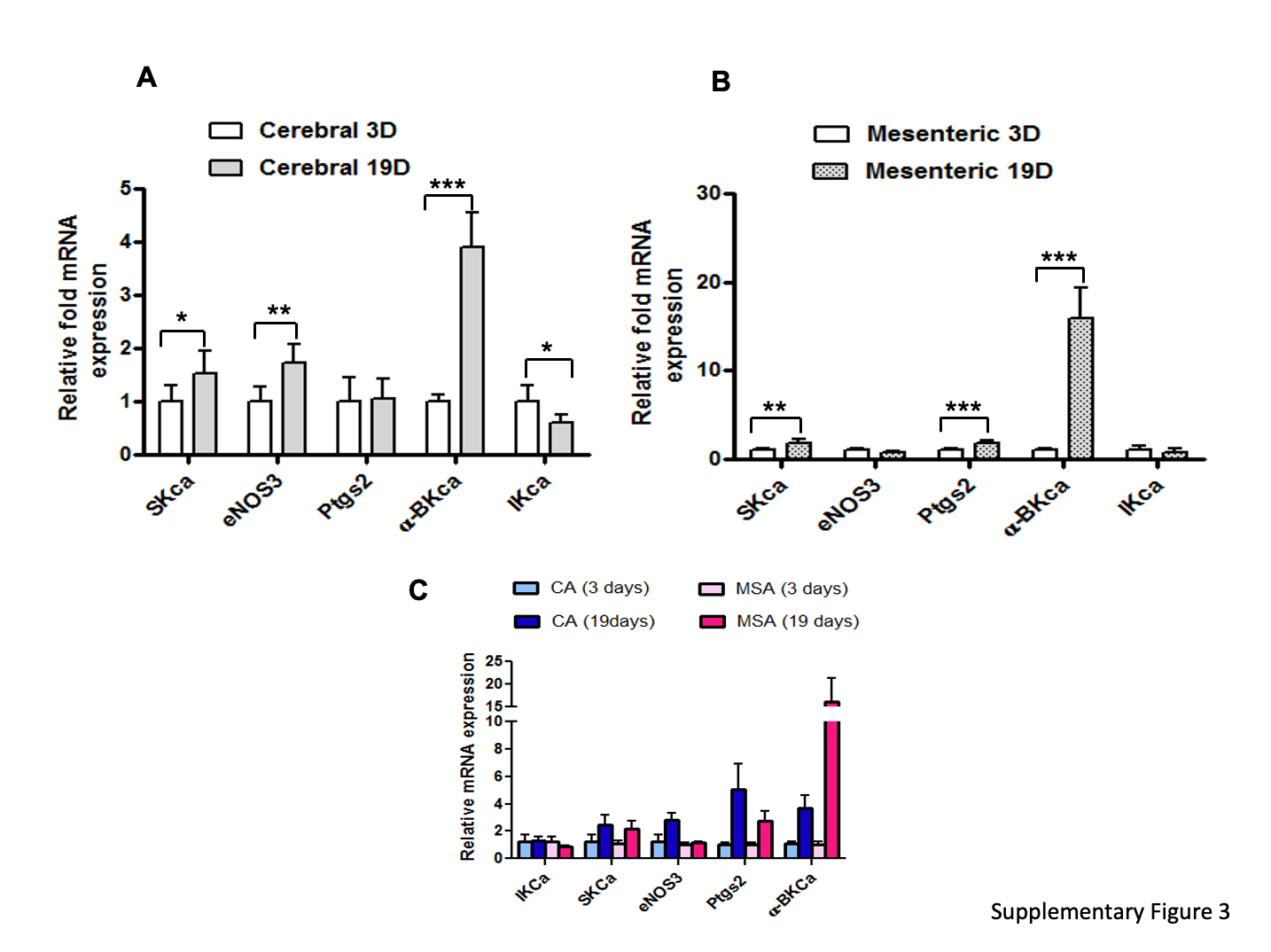

Supplement: Supplementary file 2 [file Image3.JPEG]

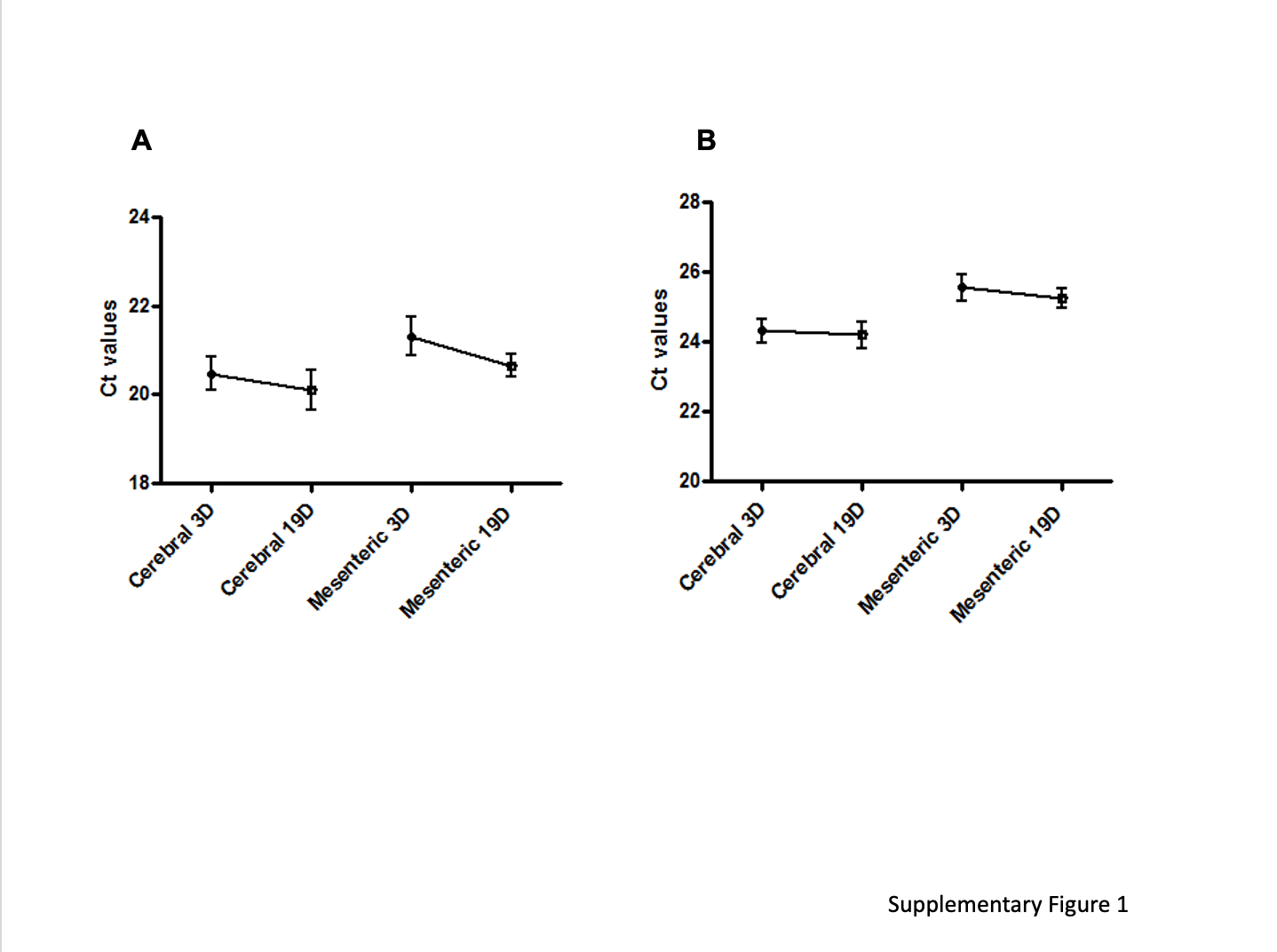

Supplement: Supplementary file 3 [file Image1.JPEG]

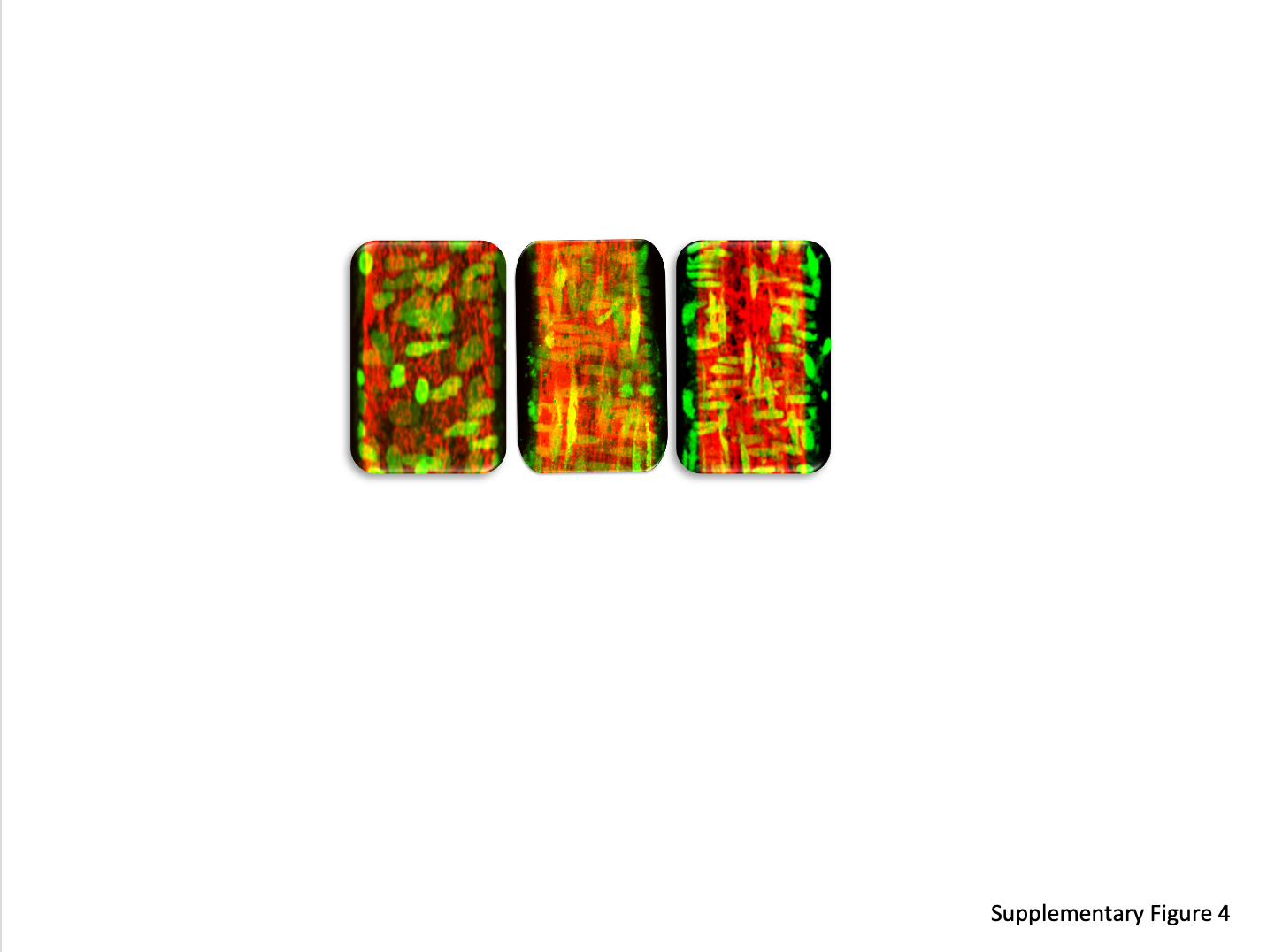

Supplement: Supplementary file 4 [file Image4.JPEG]

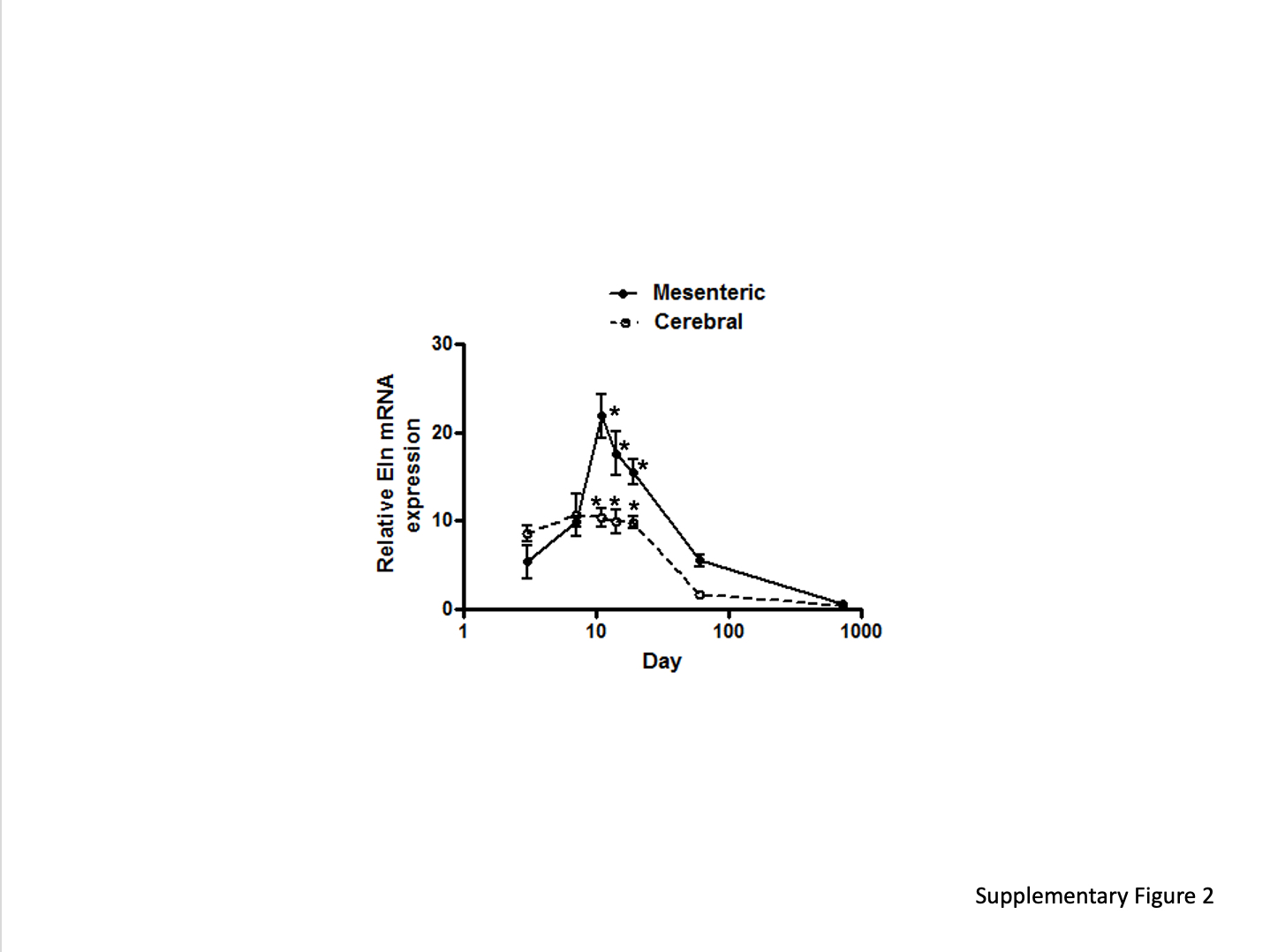

Supplement: Supplementary file 5 [file Image2.JPEG]
