# Supplementary material for: Postnatal development of extracellular matrix and vascular function in small arteries of the rat
Source: Front Pharmacol. 2023 Aug 15;14:1210128. doi: 10.3389/fphar.2023.1210128 (PMC10464837; doi:10.3389/fphar.2023.1210128)
Supplement: Supplementary file 6 [file DataSheet1.PDF]

Supplementary Table 1: *Primer sequences used in this study to identify desired extracellular matrix gene expressions for Real-time PCR using SYBER Green.*

Accession no.	Gene name	Primers sequences	PCR products (bp)
NM_031144	Actin(beta)	F: CCT CTA TGC CAA CAC AGT GCT GTC T<br>R: GCT CAG GAG GAG CAA TGA TCT TGA	128
NM_012722	Elastin	F: TTC TCC TAT CTA CCC AGG TGG<br>R: AAG ATC ACT TTC TCT TCC GG	146
NM_031825	Fibrillin1	F: GGT CAT ATC GCT GTC TCT GTA AG<br>R: CCA TTG CCA GAG AGG TTC AG	100
NM_031826	Fibrillin2	F: ACG ATG GTT ACG GAC TGG A<br>R: CAG TTA CAC TCA TAG CGA CCA G	121
NM_017061	Lysyl oxidase	F: AGA CGA TTT GCC TGT ACT GC<br>R: CAC TGA CCT TTA GAA TGT AAT TTC CG	133
NM_019386	Transglutaminase 2	F: AGC TGG AGA GCA ACA AGA GC<br>R: GCC TGG TCA TCC AGG ACT C	69

Gene names and accession numbers indicate *Rattus norveigicus* transcripts listed by NCBI webpage. F, represents forward and R, represents reverse primers.

Supplementary Table 2: *TaqMan assays used in this study to identify desired extracellular matrix gene expressions in rat cerebral and mesenteric arteries using Quantitative Real-time PCR.*

Accession number	Gene name	Gene symbol	Assay ID number
X03205	18S rRNA	18S	Hs99999901_s1
NM_031144	Actin (beta)	Actb	Rn00667869_m1
NM_017008	GAPDH	Gapdh	Rn01775763_g1
NM_012722	Elastin	Eln	Rn01499782_m1
NM_053304	Collagen 1	Col1a1	Rn01463848_m1
NM_012929	Collagen 2	Col2a1	Rn01637087_m1
NM_032085	Collagen 3	Col3a1	Rn01437681_m1
NM_001135009	Collagen 4	Col4a1	Rn01482927_m1
NM_001106710	Emilin 1	Emilin 1	Rn01469488_m1

18S rRNA was selected to verify the quality of mRNA samples. Changes in mRNA expression levels were calculated as fold changes relative to the elastin mRNA expression of an arbitrary 2 months old cerebral artery, after normalization to β-actin as a references gene. Gene symbols and accession numbers indicate *Rattus norveigicus* transcripts listed by NCBI webpage.

Supplementary Table 3: *Features of primer sets used to identify gene expression levels in cerebral and mesenteric arteries of 3 and 19 day-old postnatal rats for Real-time PCR, using SYBER Green.*

Gene symbol	Accession number	Primer sequence	Amplicon length(bp)
Actb	NM_031144	F: CCTCTATGCCAACACAGTGCTGTCT<br>R: GCTCAGGAGGAGCAATGATCTTGA	128
IK_{Ca}	NM_023021	F: GATACCCATCACGTTCCTGAC<br>R: TGTTGAACTCCAGCTTCCG	149
$SK_{Ca}3$	NM_019315	F: AAGCTATTCACCAACTGAGGG<br>R: TTTTCCAGGTCTTCACTCCG	145
eNOS3	NM_021838	F: ACCTGATCCTAACTTGCCTTG<br>R: CAGCCAAACACCAAAGTCATG	138
Ptgs2	NM_017232	F: TCAAGGGAGTCTGGAACATTG<br>R: GCTTCCCAACTTTTGTAACCG	139
α-BK_{Ca}	NM_031828	F: AAACAAGTAATTCCATCAAGCTGGTG<br>R: CGTAAGTGCCTGGTTGTTTTGG	137
Gapdh	NM_017008	F: CCATCAACGACCCCTTCATT<br>R: GACCAGCTTCCCATTCTCAG	110

Three day-old samples have been chosen as calibrator to calculate the relative fold mRNA expressions levels for each desired gene in each vasculature separately. β-actin used as a references gene to normalization. Parallel calculations have been done using GAPDH as a second housekeeping gene (see supplementary data). Gene symbols and accession numbers indicate *Rattus norveigicus* transcripts listed by NCBI webpage.
